# Holographic Brain Theory: Super-Radiance, Memory Capacity and Control Theory

**DOI:** 10.3390/ijms25042399

**Published:** 2024-02-18

**Authors:** Akihiro Nishiyama, Shigenori Tanaka, Jack A. Tuszynski, Roumiana Tsenkova

**Affiliations:** 1 Graduate School of System Informatics, Kobe University, 1-1 Rokkodai, Nada-ku, Kobe 657-8501, Japan; tanaka2@kobe-u.ac.jp; 2 Aquaphotomics Research Department, Graduate School of Agricultural Science, Kobe University, 1-1 Rokkodai, Nada-ku, Kobe 657-0851, Japan; 3 Yunosato Aquaphotomics Lab, Hashimoto 648-0086, Japan; 4 DIMEAS, Politecnico di Torino, Corso Duca degli Abruzzi 24, I-1029 Turin, Italy; 5 Department of Physics, University of Alberta, 11335 Saskatchewan Dr NW, Edmonton, AB T6G 2M9, Canada; 6 Department of Data Science and Engineering, The Silesian University of Technology, 44-100 Gliwice, Poland

**Keywords:** holography, super-radiance, memory, reservoir computing, control theory

## Abstract

We investigate Quantum Electrodynamics corresponding to the holographic brain theory introduced by Pribram to describe memory in the human brain. First, we derive a super-radiance solution in Quantum Electrodynamics with non-relativistic charged bosons (a model of molecular conformational states of water) for coherent light sources of holograms. Next, we estimate memory capacity of a brain neocortex, and adopt binary holograms to manipulate optical information. Finally, we introduce a control theory to manipulate holograms involving biological water’s molecular conformational states. We show how a desired waveform in holography is achieved in a hierarchical model using numerical simulations.

## 1. Introduction

The human brain is one of the most complex and fascinating structures in the world. The question about the molecular mechanisms involved in the human brain’s functioning has been of great interest to not only neurophysiologists but also information scientists, biophysicists and psychologists for decades. While much is known about the types, organizational structure of brain cells, and their electrical and biochemical activities, much less is known about such enigmatic issues as where our memories are stored, or which molecular mechanisms are involved in information processing by brain cells. Speculations about these molecular mechanisms behind cognition abound. Recently, heated debates centered on the possibility that at least some cognitive functions operate at a quantum level.

Nobel-Prize winning neuroscientist Eric Kandel discovered that as we learn, chemical signals change the structure of synaptic connections [1]. He also showed that short-term and long-term memories are formed by electrical signals transmitted across synapses in a process called long-term potentiation (LTP). Specifically, repetitive pre-synaptic stimulation increases post-synaptic sensitivity and hence strengthens synapses. This is aptly expressed by saying that neurons that fire together, wire together. While LTP has provided a glimpse into the nature of learning and memory, the issue appears to be much more complicated. First, while long-term memory endures, LTP does not make permanent changes to synaptic strength but decays over hours to months. Second, LTP-based memory models suffer from the loss of signal fidelity. Third, memory requires the assimilation of information across different sensory inputs involving huge neural circuits that need to be somehow integrated. Finally, external stimuli are associated within their context, so that new experiences are influenced by the current context, previous experiences, and even anticipation of the future. To explain this with only synaptic-based computing may easily exceed the capabilities of the brain. This suggests that another level of computation in the brain is needed for learning and memory functions.

Questions such as whether classical physics is adequate to properly explain the complexity of human mind are hotly debated with sharp division lines between supporters of classical neurophysiology and their opponents who strongly favor quantum approaches to consciousness as the ultimate explanation of how the brain really works when it creates conscious experience. One of the first attempts to describe the brain using quantum physics was made by Ricciardi and Umezawa in 1967 [2]. Based on experimental observations of brain activity they proposed that the brain can be excited into particular quantum states by stimuli from the external environment. Thus, information can be thought of as being coded into the brain as quantum excited states representing short-term memory. This code would then be later on transferred to the lowest quantum energy state by means of a Bose–Einstein condensation accounting for learning and long-term memory represented by macroscopic quantum fields. Memory in the brain is stored in a diffused unlocalized domain of a quantum field theoretical vacuum acting like a single entity of the Bose–Einstein condensate as that in superconducting media [3]. This model, called quantum brain dynamics (QBD), proposes that brain functions are manifestations of spontaneous symmetry breaking in the dynamical state of the brain [4]. It was later extended to view the brain as a hybrid physical system with the first part consisting of the classical electrochemical interactions in neurons, and the second being the macroscopic quantum state responsible for the creation and maintenance of memory. This was later fleshed out by Del Giudice and co-authors [5,6,7]. Jibu and Yasue included the dipolar field of water molecules in the brain interacting with the internal electromagnetic field [4]. An extension of QBD included microtubules, as well as dendritic and neural networks. Microtubules were predicted to form nonlinear optical devices akin to lasers creating electromagnetic field inside their hollow core in a process termed ‘super-radiance’. The optical computing proposed to occur in these microtubule networks was viewed to provide the basis for cognitive functions.

What is the physical mechanism of memory involving our subjective experience in the brain? It is still an open question. We know several features of memory produced in the human brain, which are distinguished from computer memory [8]. We recall a song or a melody in the forward direction, not backward, (sequential patterns). When part of information is given, we recall the whole memory auto-associatively (auto-associative recalling). Memory is processed in a hierarchy in the neocortex, namely in regions V1, V2, ⋯, V5, for visual processing (storage in a hierarchy). Memory is stored in patterns in an invariant form. In taking a cup and drinking tea, all motions are different each time, but we recognize them as a single movement. Memory has diffused nonlocal features and is not localized in a particular region of the brain [9]. Even if part of a brain is damaged, memory is recalled by remaining undamaged areas [10], which is known as equipotentiality [11]. Whether or not particular memory is lost depends on the magnitude of widespread lesions in the brain, known as the mass action principle [9]. Memory is robust against damages done to the brain. To explain these features of memory, Pribram proposed the holographic brain theory as a candidate of theory of memory and perception [12,13]. Holography, a technique to record 3-dimensional information on medium invented by Gabor [14], can describe various features on memory in a brain as listed above. Simultaneous optical information processing can be achieved due to super-position of optical waves even in classical holography. Holonomy (quantum holography) adopted by Pribram provides the useful properties that a great amount of information is stored within a small region representing patches of dendritic receptive fields in ‘cellular’ phase space, and that though the information in the patch is entangled, cooperative information processing among patches is achieved coherently, and decoherence can induce the localization of the process [15]. Cavaglia et al. recently proposed a holographic paradigm in the brain involving neuronal membrane dipole oscillations with quantum coherence [16]. The collaborators of Pribram were Jibu and Yasue who proposed the concrete degrees of freedom in Quantum Brain Dynamics (QBD), that is Quantum Field Theory (QFT) of the brain, namely water electric dipole fields and photon fields [4,17,18,19,20,21]. Quantum Field Theory (QFT) of the brain is one of the hypotheses expected to explain formation of memory in the brain. QFT, which is distinguished from quantum mechanics, is applied to describe both microscopic degrees of freedom in quantum mechanical sense and macroscopic matter in the sense of classical physics [22]. This is called the Jibu–Yasue approach, which is distinguished from the Penrose–Hameroff approach to consciousness [23,24].

The QBD theory is originated with the monumental work by Ricciardi and Umezawa in 1967 [2]. It is further developed by Umezawa, Stuart and Takahashi in 1978–1979 [25,26]. In 1968, Fröhlich proposed the Bose–Einstein condensation an biological systems with quantum coherence involving long-range correlations, called Fröhlich condensation [27,28]. In 1976, Davydov and Kislukha proposed a coherent dipolar solitary wave solution propagating along the one-dimensional chain of alpha-helix structures in protein molecules such as protein filaments, called the Davydov solution [29]. In QFT, a coherent solitary wave propagates as a localized degree of freedom storing and transferring energy without loss due to thermal effects [3]. The Fröhlich condensation and the Davydov soliton solution represent static and dynamic features, respectively, emerging in a non-linear Schrödinger equation with an equivalent Hamiltonian [30]. In 1980s, Del Giudice et al. proposed to use a quantum field theoretical method in the description of dynamics of biological systems [31,32,33,34]. They investigated the laser-like behaviors in Quantum Electrodynamics (QED) of water dipoles and photons by considering water molecules’ rotational degrees of freedom [33]. Within the QED framework, Preparata argued about coherent super-radiance solutions which were first introduced by Dicke in 1954 [35,36,37]. Recently, Keppler adopted the QED theory formulation introduced by Preparata to investigate the feasibility of coherent domains of glutamates in the human brain [38]. A recent experimental study by Kerskens suggests the presence of quantum entanglement of excited states emerging in a brain [39]. Dotta et al. showed that photon emission from the head increased while subjects imagined light in a very dark environment [40]. Kauffman et al. proposed that measurements by our mind convert possibilities manifested in quantum superpositions of states to actual events [41,42]. In QBD, memory corresponds to the vacua emerging in the breakdown of rotational symmetry of quantum degrees for freedom [4]. Therefore, it is a logical conclusion to suggest integration of QBD with the holographic brain theory [19,43]. Or, we might adopt a dissipative quantum brain theory with squeezed coherent states of Nambu–Goldstone bosons in open systems [44], which is equivalent to fractal functional representations as earlier proposed by Vitiello [45,46].

The main criticism levelled at QBD is about the role of thermal decoherence phenomena of the proposed quantum states [47]. It was claimed that quantum coherence in the brain cannot be maintained since the brain system is too warm, too wet and too noisy. The order of magnitude of the decoherence time is $10^{-20}$ s as estimated by Tegmark. However, his analysis has several major problems. First, he adopts the mass of a water molecule as ∼18×940 MeV. To estimate the macroscopic order of water rotational degrees of freedom in QBD, we should use the inverse of the moment of inertia ∼4 meV or the mass of polaritons formed from water fields and photon fields. Moreover, he adopts a strange procedure where time scales for decoherence are divided by the number of relevant Na ions $10^{6}$. In addition, we need to consider the brain as an open system, which is constantly supplied with metabolic energy for it stay alive. In the Fröhlich model, the physical system is connected with an energy supply and a heat bath [30]. In the open system, we should investigate the balance of decoherence and error corrections for quantum coherence in the flow of energy from the energy supply through the physical system to the heat bath. His approach for decoherence lacks these types of analysis and hence the resultant estimates of the decoherence times are unrealistically small.

We should also comment on the reasons why we focus on water molecules. In the main stream of neuroscience and physiology, most researchers investigate constituent elements of the human brain, such as neurones, proteins, DNA, ions, and so on, for physiological processes, and consider water molecules as merely an inert solvent medium. In this view, water plays a role of a supporting actor, not one of the main actors. However, to achieve long-range correlations in the whole brain, we need degrees of freedom present in the entire brain. Lashley indicated an unsolved problem for nervous system organization and masses of excitations in the limited paths of nerve cells, which is Referred to as Lashley’s dilemma [48]. The most likely candidates for such degrees of freedom are photons and water molecules. Quantum Brain Dynamics adopts photons and water as main actors covering the whole brain involving their internal organization in the formation of our conscious experiences. Brain is a mixed system of classical neurons and glial cells on the one hand, and quantum degrees of freedom of photons and water molecules on the other hand. The latter obey the laws of quantum field theory (QFT). In QFT, we adopt spontaneous symmetry breaking (SSB) representing macroscopic order in a physical system. Order is maintained by long-range correlations by massless Nambu–Goldstone quanta emerging in SSB. We can adopt macroscopic order in QFT in the brain to describe masses of excitations. Moreover, water is also an amplifier of dynamical effects of charged ions, and the dipolar degrees of freedom of the cytoskeleton. Sizeable dipoles affect orientations of water dipoles [33]. Electric dipoles of tubulin dimers in a microtubule affect surrounding water dipoles due to their dipole-dipole interactions. Conversely, surrounding water molecules as a group also affect the dynamics of the cytoskeleton, especially in neutrons where microtubules form parallel bundles. Water dipoles and constituents of ions, proteins, and so on, are hence dynamically correlated. It should also be stressed that the cerebrospinal fluid is a high electric conductivity medium, very well suited for the transmission of electromagnetic signals across all areas of the brain. This, local events in the constituents might be readily and faithfully transferred via water dipoles across the whole brain. A recent experimental study of microtubule excitations has shown the presence of quantum effects on a nanometer and nanosecond scale [49].

Whether or not our brain adopts the language of holography might be investigated by manipulating holograms by external stimuli. A recently reported experiment for invasive stimulation to manipulate our visual subjective experience was described in [50], for example. We prefer non-invasive methods. Non-invasive neural stimulation methods have been developed over several decades [51], and originated from a seminal work by Barker as transcranial magnetic stimulation (TMS) [52]. Transcranial electric stimulation with direct current [53] and alternating current [54], photonic methodology with near-infrared photons [55,56], and an ultra-sound method [57,58,59] have been also developed and applied to treat neuropsychiatric diseases. Our approach in this paper is based on non-invasive manipulation of holograms within the holographic brain theory. We adopt reservoir computing or morphological computation [60,61,62] as a control theory of holograms.

The aim of this paper is to derive a super-radiance solution in the holographic brain theory, estimate memory capacity of a neocortex and to introduce a control theory to manipulate holographic memory involving our subjective experiences. First, we adopt the QED theory with non-relativistic charged bosons, corresponding to the holographic brain theory proposed by Pribram [13]. The QED theory corresponds to a model of water molecules’ molecular conformational states involving super-radiant coherent photon emissions expected to achieve interference patterns in holography. Next, using the wavelength of super-radiant emission, we can estimate memory capacity in a holographic brain model. Finally, we propose a control theory manipulating holograms by adopting morphological computational approach [62]. We adopt a hierarchy of multiple layers as a model of neocortex covered by cerebrospinal fluid, dura and skull. We find that binary holograms involving step-function-like distributions of density distribution of water molecular conformational states are realized by external input electromagnetic fields propagating through multiple layers in a hierarchy.

This paper is organized as follows. In Section 2, we derive a super-radiance solution in the framework of QED corresponding to holographic brain theory by Pribram. In Section 3, we show holographic aspect in QED and derive memory capacity of a brain neocortex. In Section 4, we derive time-evolution equations in QED in background field gauge and show how a desired waveform (hologram) of coherent charged Bose fields is achieved in numerical simulations. In Section 5, our results are discussed. In Section 6, concluding remarks and perspectives are provided. We adopt the natural unit with the light speed and the Planck constant *ℏ* set to be 1. The metric tensor is set to be $\eta_{\mu \nu }=\mathrm{diag}\left(1,-1,-1,-1\right)$ with space-time subscript μ,ν=0,1,2,3 and spatial subscript i,j=1,2,3.

## 2. Super-Radiance Solution

**Figure 1:**
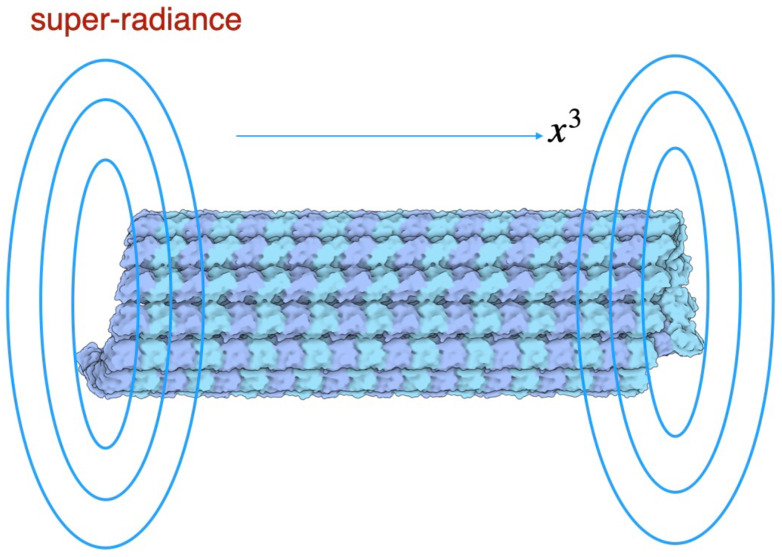
Super-radiant emission from a microtubule.

In this section we show a super-radiance solution in Quantum Electrodynamics (QED) with non-relativistic charged bosons (a model of water molecular conformational states) as a resource of super-radiant coherent light to achieve hologram memory in a brain. We adopt water molecules inside microtubules as sources of super-radiance as shown in Figure 1.

Derivation of super-radiance solution is given in Appendix A. The amplitude of electric field $E_{3}$ is,

(1) $$\begin{array}{c} E_{3}=\frac{g\Omega^{2}N}{4\pi }{\left[\cosh\left(\frac{x^{0}-\tau_{0}}{\tau_{R}}\right)\right]}^{-1}, \end{array}$$

where *g* represents the coupling defined in Appendix A, *N* represents the number of water molecules, Ω represents energy difference between the ground state and 1st excited state in two-energy level approximation, $x^{0}$ represents time, $\tau_{R}$ represents,

(2) $$\begin{array}{c} \tau_{R}=\frac{2\pi }{g^{2}\Omega N}, \end{array}$$

with $\tau_{0}=-\tau_{R}\ln\tan\frac{\theta_{0}}{2}$ (definition of $\theta_{0}$ is in Appendix A). Since *N* molecules in the 1st excited state decay with $\tau_{R}∼1/N$ time scales, the intensity of coherent light is the order of $N^{2}$. We find the solution of the flash light, namely super-radiance representing cooperative spontaneous emission of light via a microtubule.

## 3. Memory Capacity

In this section, we evaluate the memory capacity for neocortex for a given wavelength, and introduce the theory of computer generated holograms. Holographic aspect is shown in Appendix B.

We shall estimate limitations of hologram memory storage in the neocortex. The limitation is determined by whether or not binary signals can be divided in changing radiation angles of coherent laser lights [63]. The limitation of 2-dimensional holograms is $\frac{4}{\lambda^{2}}$ with wavelength of laser light λ. When holograms have a finite thickness *d* of the neocortex, the factor $\frac{n_{r}d}{\lambda }$ with the refractive index of holograms $n_{r}$ is multiplied. The capacity per unit area is calculated as,

(3) $$\begin{array}{c} \mathrm{Capacity}\;\mathrm{per}\;\mathrm{unit}\;\mathrm{area}=\frac{4n_{r}d}{\lambda^{3}}=8\times 10^{10}\;\mathrm{bit}/{\mathrm{mm}}^{2}, \end{array}$$

where we have used $n_{r}=1.3$, d=2mm for neocortex and an example of wavelength λ=500nm. Multiplying the area of the **neocortex** 2500 cm^2^, we arrive at

(4) $$\begin{array}{c} \mathrm{Capacity}=2.5\;\mathrm{PB}. \end{array}$$

We shall compare the above value with the memory capacity estimated in conventional neuroscience. The number of synapses is $250\times 10^{12}$ per brain. When we memorize information 1 bit per synapse, the capacity of memory storage for **whole brain** is,

(5) $$\begin{array}{c} \mathrm{Capacity}\;\mathrm{in}\;\mathrm{conventional}\;\mathrm{neuroscience}=30\;\mathrm{TB}. \end{array}$$

Memory capacity in the holographic brain theory is huge compared with its capacity estimated using conventional neuroscience.

**Figure 2:**
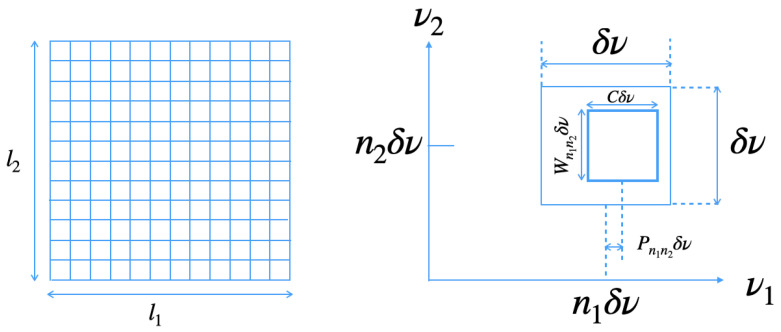
Method for computer-generated binary holograms. (**Left**: Square cells, and **Right**: Hole in the cell).

Finally, we denote computer-generated binary holograms introduced by Lohmann, Brown and Paris [64,65,66]. We introduce a brief summary of how binary figural information is recorded in holograms in their method. We adopt binary gradation of holograms with high and low transmittance. First, we divide holograms into square cells as depicted in the left of Figure 2. Next, we make rectangular holes in $\left(n_{1},n_{2}\right)$ cell involving 2-dimensional $\nu_{1}$-$\nu_{2}$ plane in the right of Figure 2. The center of the rectangular hole is the position $\left(\left(n_{1}+P_{n_{1}n_{2}}\right)\delta \nu ,n_{2}\delta \nu \right)$. The area of the hole is represented by,

(6) $$\begin{array}{ccc} |\nu_{1}-\left(\left(n_{1}+P_{n_{1}n_{2}}\right)\delta \nu \right| & \leq & \frac{C}{2}\delta \nu , \end{array}$$

(7) $$\begin{array}{ccc} |\nu_{2}-n_{2}\delta \nu | & \leq & \frac{W_{n_{1}n_{2}}}{2}\delta \nu , \end{array}$$

for high transmittance. When the binary figural information (as a picture) to be reconstructed is given by $U(x^{1},x^{2})$ and its Fourier transformation $\tilde{U}\left(n_{1}/X^{1},n_{2}/X^{2}\right)\propto B_{n_{1}n_{2}}\exp\left(i\Phi_{n_{1}n_{2}}\right)$ with the size of the picture $X^{1}\times X^{2}$ is given, we set,

(8) $$\begin{array}{ccc} W_{n_{1}n_{2}} & = & B_{n_{1}n_{2}}, \end{array}$$

and,

(9) $$\begin{array}{ccc} 2\pi NP_{n_{1}n_{2}} & = & \Phi_{n_{1}n_{2}}, \end{array}$$

where N is an arbitrary integer. We shall adopt binary holograms to manipulate optical signal transfer in a brain. In the next section, we see how rectangular holes are constructed in a control theory based on morphological computation.

## 4. Control Theory

**Figure 3:**
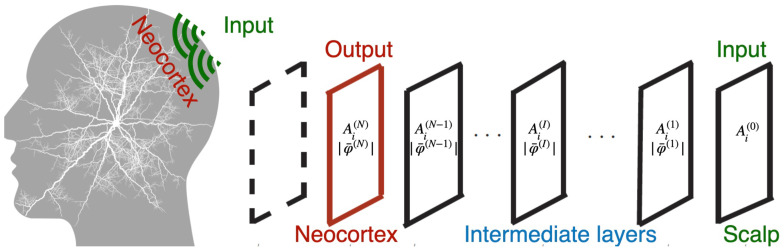
Quantum fields in a hierarchy involving input 0th layer, intermediate I=1,2,⋯,N−1th layers and output *N*th layer.

In this section, we use a control theory applied to the holographic brain theory given in Appendix C and show how holograms involving density distributions of charged bosons are manipulated by external input functions. We adopt quantum fields in a hierarchy given in Figure 3. Time-evolution equations are given in Equations (A82)–(A86) with (A87). The input functions are calculated by Equations (A88) and (A89) for given target functions $A_{1,\mathrm{target}}$ and $A_{2,\mathrm{target}}$.

We shall consider N=4 layers. We set a 2-dimensional spatial lattice for each layer in Figure 3 by $x^{i}=-N_{s}a_{s},-\left(N_{s}-1\right)a_{s},\cdots ,n^{i}a_{s},\cdots ,\left(N_{s}-1\right)a_{s},N_{s}a_{s}$ with discrete integer $n^{i}$ for $x^{i}$ with i=1,2, lattice size $2N_{s}=128$ and lattice spacing $ma_{s}=1.0$ scaled by mass *m*. We adopt periodic boundary conditions for spatial coordinates $x^{i}$. We set time step $a_{t}$ with $a_{t}/a_{s}=0.001$. We set coupling e=0.3, transmittance $v/m^{2}=0.8$, damping factor γ/m=0.2, effective mass added $M^{2}=2m^{2}$, and the damping factor for input functions $\gamma_{2}/m=0.01$. To investigate the time-evolution of the prepared system in a hierarchy, we adopt 4th-order Runge–Kutta method.

We set the desired target function $A_{1,\mathrm{target}}$ and $A_{2,\mathrm{target}}$ scaled by mass *m* as,

(10) $$\begin{array}{ccc} A_{1,\mathrm{target}}=u_{1}^{\left(N\right)}\left(n^{1},n^{2}\right) & = & \left\{\begin{array}{cc} 0.1\frac{n^{1}+N_{s}}{N_{s}-N_{1}} & \left(n^{1}\leq -N_{1}\right)\\ -0.1\frac{n^{1}}{N_{1}} & \left(-N_{1}<n^{1}<N_{1}\right)\\ 0.1\frac{n^{1}-N_{s}}{N_{s}-N_{1}} & \left(N_{1}\leq n^{1}\right) \end{array}\right. \end{array}$$

(11) $$\begin{array}{ccc} A_{2,\mathrm{target}}=u_{2}^{\left(N\right)}\left(n^{1},n^{2}\right) & = & \left\{\begin{array}{cc} 0.1\frac{n^{2}+N_{s}}{N_{s}-N_{2}} & \left(n^{2}\leq -N_{2}\right)\\ -0.1\frac{n^{2}}{N_{2}} & \left(-N_{2}<n^{2}<N_{2}\right)\\ 0.1\frac{n^{2}-N_{s}}{N_{s}-N_{2}} & \left(N_{2}\leq n^{2}\right) \end{array}\right. \end{array}$$

with $N_{1}=30$, $N_{2}=20$ and $u_{1}^{\left(N+1\right)}=u_{2}^{\left(N+1\right)}=0$. The $A_{1,\mathrm{target}}$ and $A_{2,\mathrm{target}}$ represent distributions of 3 polygonal lines.

**Figure 4:**
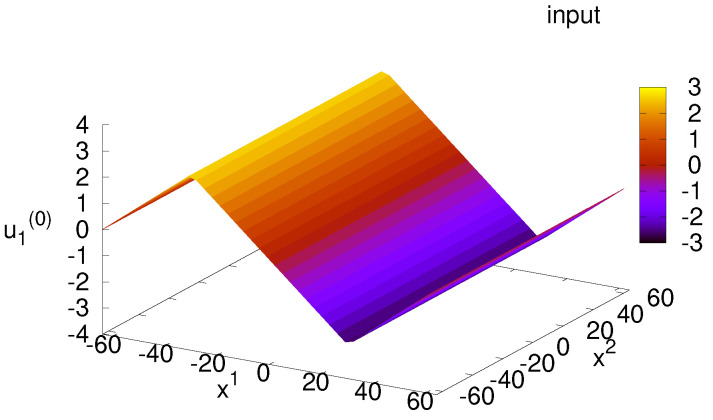
Distribution of input function $u_{1}^{\left(0\right)}$ for N=4.

The input function $A_{1}^{\left(0\right)}=u_{1}^{\left(0\right)}$ at $mx^{0}=0.0$ for N=4 is depicted in Figure 4. We find distribution with polygonal lines in $u_{1}^{\left(0\right)}$ since terms $\partial_{2}^{2}u_{1}^{\left(n\right)}$ and $\partial_{1}\partial_{2}u_{2}^{\left(n\right)}$ in Equation (A88) is zero for given target function in Equation (10). The maximum value is approximately 3 due to the factor $0.1\times \frac{{\left(M^{2}/m^{2}\right)}^{4}}{v^{N}}=0.1\times 2^{4}/0.8^{4}=3.9$ in deriving input function $u_{1}^{\left(0\right)}$.

We set initial conditions $|{\bar{\varphi }}^{\left(n\right)}\left(x^{0}=0,x\right)|=1.0$ scaled by mass *m*, $A_{1}^{\left(n\right)}=A_{2}^{\left(n\right)}=0$ and $E_{1}^{\left(n\right)}=E_{2}^{\left(n\right)}=0$ with $A_{0}^{\left(n\right)}=0$ (n=1,2,⋯,N). We fix $A_{1}^{\left(N+1\right)}$, $A_{2}^{\left(N+1\right)}$, $E_{1}^{\left(N+1\right)}$ and $E_{2}^{\left(N+1\right)}$ to zero for any time point.

**Figure 5:**
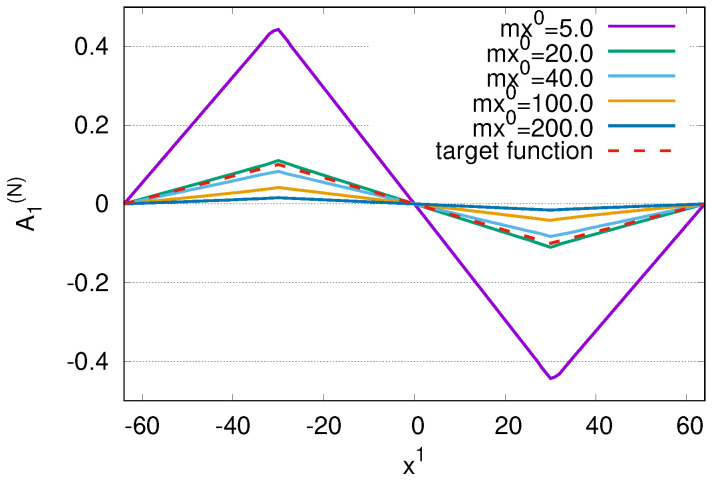
Time−evolution of distributions $A_{1}^{\left(N\right)}\left(x^{0},x^{1},x^{2}=0\right)$ for N=4 at $mx^{0}=5.0$, 20.0, 40.0, 100.0 and 200.0 with target function $A_{1,\mathrm{target}}$.

In Figure 5, we depict the time-evolution of distributions $A_{1}^{\left(N\right)}\left(x^{0},x^{1},x^{2}=0\right)$. At $mx^{0}=0.0$, we begin with zero $A_{1}^{\left(N\right)}$. At $mx^{0}=5.0$, we find polygonal lines with its maximum value larger than the maximum value of target function. The distribution $A_{1}^{\left(N\right)}$ approaches the target function at $mx^{0}=20$ due to damping term $-\gamma E_{1}^{\left(N\right)}$ in Equation (A82). At later times $mx^{0}=40$, 100 and 200, the distribution $A_{1}^{\left(N\right)}$ decays in passage of time since the input function $A_{1}^{\left(0\right)}$ decays exponentially due to factor $\exp(-\gamma_{2}x^{0})$ with $\gamma_{2}=0.01$. At $mx^{0}=200=2/\gamma_{2}$, the $A_{1}^{\left(N\right)}$ converges to zero due to damping term $-\gamma E_{1}^{\left(N\right)}$ in Equation (A82) and damping of input function $A_{1}^{\left(0\right)}$. Using distributions of polygonal lines, we can manipulate density distributions of charged Bose fields $|{\bar{\varphi }}^{\left(N\right)}{|}^{2}$.

**Figure 6:**
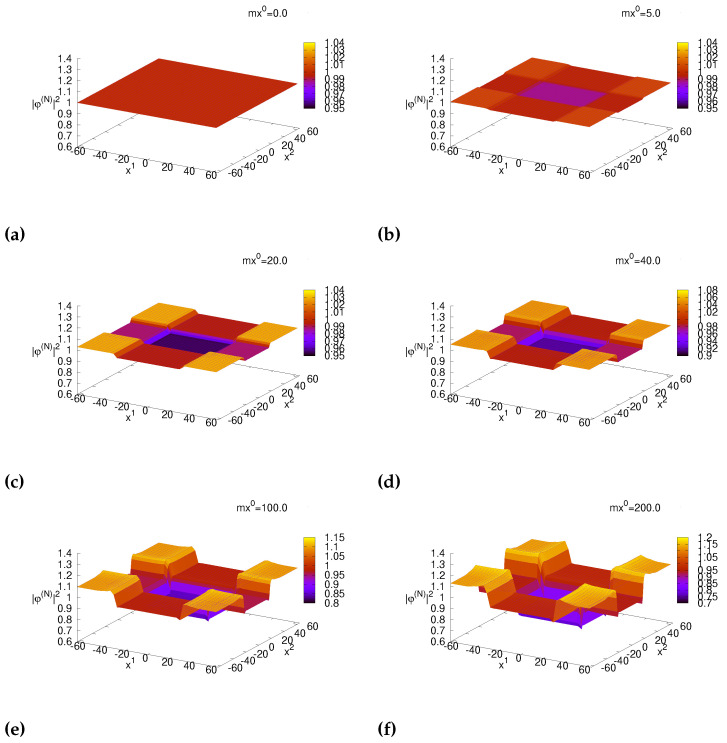
Distribution of $|{\bar{\varphi }}^{\left(N\right)}{\left(x\right)|}^{2}$ at (**a**) $mx^{0}=0.0$, (**b**) $mx^{0}=5.0$, (**c**) $mx^{0}=20.0$, (**d**) $mx^{0}=40.0$, (**e**) $mx^{0}=100.0$ and (**f**) $mx^{0}=200.0$.

We show the time-evolution of density distributions of charged Bose fields corresponding to holograms in Figure 6. The initial distribution represents homogeneity with initial value 1.0 as shown in Figure 6a. As time goes by, distributions in the area $-N_{1}a_{s}<x^{1}<N_{1}a_{s}$ with $N_{1}=30$ and $-N_{2}a_{s}<x^{2}<N_{2}a_{s}$ with $N_{2}=20$ start decreasing. The decrease is small (around 1.3%) at $mx^{0}=5.0$ in Figure 6b compared with the initial value 1.0 in Figure 6a, and distributions in the area are nearly homogeneous. At $mx^{0}=20.0$ when $A_{1}^{\left(N\right)}$ is near target function $A_{1,\mathrm{target}}$, the decrease the area is approximately 4.5%. At $mx^{0}=40.0$ the decrease is near 9.1%, and the shape is approximately step-function. The decrease is 16% is at $mx^{0}=100.0$. The decrease is 21% at $mx^{0}=200.0$, but step-function is slightly distorted. The deviation of the total number of charged bosons from its initial value is less than $10^{-6}$% in the output layer (N=4) according to our numerical simulations, representing the number conservation on lattice simulations. Step-function-like rectangular holes are adopted as binary holograms in the holographic brain theory.

## 5. Discussion

The existence of a “quantum mind” could bridge the gap between mind and brain through quantum mechanics. The quantum degrees of freedom of the mind would represent a separate dynamical sector and would determine the state of the brain at a quantum level (the quantum brain), which could access the sensory inputs from the various receptors in addition to the classical physiological mechanism for the remaining degrees of freedom of brain dynamics. Traversing in the opposite direction, the quantum mind would affect the macroscopic classical brain and result in directed actions commanded by the quantum mind. The profound insights given by the laws of quantum physics can aid in solving the mystery of consciousness in several ways. Quantum physical systems possess both wave and particle properties, which is referred to as the particle-wave duality principle. As waves, quantum systems act as a whole. The boundary of a quantum system, which is described by a wave function, is somewhat blurred and there is no hard and fast rule how a system ceases to be quantum and starts being classical as its size increases. As waves, these quantum systems are extended over physical space and occupy multiple physical states simultaneously. This extension can be reduced to a single classical state through interaction with its environment or spontaneously, which is called a wave function collapse. While quantum systems operate deterministically in the Schrödinger equation governing the wavefunction of the system and hence the occupation probabilities for individual quantum states, they evolve not deterministically due to the random nature of the measurement and decoherence processes. Hence, quantum systems exhibit a blend of deterministic evolution and probabilistic measurement outcomes, making them fundamentally different from classical deterministic systems. Also, due to the Heisenberg uncertainty principle, they are never completely knowable. These are attractive properties for implementation in the context of understanding the human mind.

But how a quantum mind could perceive the macro-world is yet another mystery altogether. After all, information also travels from our senses to our consciousness. The brain gathers sensory input data, processes this information, after which an answer is consciously decided on, and transformed into action such as producing sound waves using one’s voice. These activations then cause changes in the quantum state of individual atoms. A quantum mind would have to:

1. Register quantum level changes in neurons caused by sensory inputs,
2. Decipher this information and translate into high-level concepts,
3. Make a decision on the quantum level about the reaction to the inputs,
4. Encode that decision back into the quantum level to produce a macroscopic effect.

In this paper, we have adopted Quantum Electrodynamics (QED) with non-relativistic charged Bose fields corresponding to the holographic brain theory by Pribram. Beginning with the Lagrangian density, we have shown a super-radiance solution around neuronal microtubules in the brain, and provided a holographic aspect within the QED framework with estimates of the memory capacity and the introduction of binary holograms. We also introduced a control theory of binary holograms using morphological computation. Manipulating holograms by external electric fields, we might be able to affect our memory and subjective experiences. When super-radiant waves from microtubules are imposed on binary holograms manipulated by external electric fields, optical information can be reconstructed and artificial memory induced by external electromagnetic fields might be recalled with our subjective experiences.

Quantum coherence in microtubules might provide saltatory coupling between spines and induce saltatory conduction in axons via solitary waves covering a longer range for maintaining coherence, as proposed independently by Davydov, Adey, and Pribram [3,13,15,67]. Quantum computation within the brain was predicted by Penrose and Hameroff to occur within neuronal microtubules [24]. Tubulin is the building block of microtubules and it has been considered to carry biological quanta of information, qubits. Tubulin proteins are envisaged to interact with their neighboring tubulin molecules in a microtubule and by doing so perform quantum computations using entanglement effects. The biological conditions in the brain, including synaptic activity, are considered to influence the quantum computations thus orchestrating the collapse of such qubits giving rise to a conscious event when that happens. Water can then be a key element for amplification of local events for qubits to macroscopic phenomena covering the whole brain. The QFT of water and photons can provides a framework in both microscopic and macroscopic properties in a brain.

We have adopted microtubules as coherent super-radiant light sources in the holographic brain theory. Super-radiance suffers from the dephasing effect and as a result cooperative property of super-radiance is weakened [68] due to asymmetry of van der Waals interactions among atoms or dipoles, which correspond to water molecular conformational states in the present case. To overcome the dephasing effect, the geometry of the system composed by water molecules plays a significant role. Arranging water molecules on a ring, van der Waals interactions induce symmetric property and dephasing effect is expected to diminish. Checking the shape of microtubules, they are found to have cylindrical structures involving rings of tubulins. The cylindrical structures are made use of to arrange water molecules on rings to diminish the dephasing effect. We should also emphasize the energy supply to microtubules by mitochondria in a cell such as a neutron [69,70]. We can consider at least three types of energy supply to microtubules, namely energy released from hydrolysis of guanosine triphosphate (GTP), energy supplied from the motion of motor proteins (dynein and kinesin), and the Waste-product energy released from mitochondria. In these forms of energy supply, wasted energy from mitochondria is the largest, that is on the order of $10^{-13}\;W$. Energy from mitochondria might be used as a supply for emission of super-radiant light of microtubules. Microtubules work as single mode waveguides with a cutoff wavelength 21 nm, in brief they should be able to guide light from strong ultra-violet to near-infrared region. Mitochondria with filamentous structures act like lasers dependent on their metabolic state [71].

Memory capacity is estimated by using the wavelength of coherent super-radiant waves. Water is an ultimate light source inducing coherent lasers [33]. Water is also an ultimate sensor of photons [72]. Coherence in water was experimentally determined using Near-Infrared spectroscopy [73]. Holograms of water media might be achieved by interference patterns of object and reference waves irradiated by water media around microtubules, and other structures in the brain. Using wavelength λ=500 nm for visible light, memory capacity is 2.5 PB which is much larger than 30 TB in a whole brain estimated by the total number of synaptic connections as used by conventional neuroscience. Even if we adopt near-infrared regions with wavelength λ = 1500 nm, the capacity is ∼0.1 PB since memory capacity is proportional to $1/\lambda^{3}$. Although Vitiello has estimated a huge memory capacity in QBD using squeezed coherent states for dissipative Quantum Field Theory [44], we have shown the above values for the neocortex in our holographic approach. We should compare our analysis with the Landauer principle [74,75,76,77]. The Landauer principle suggests that the recording and erasure of one bit of information require minimum energy $k_{B}T\ln2$ with the Boltzmann constant $k_{B}$ and temperature *T*. Physiological temperature T=310 K indicates $k_{B}T\ln2=20$ meV. In case we adopt super-radiant waves with wavelength λ=500 nm, the energy of the photon used to record holographic images is 2.4 eV. This energy scale is much larger than $k_{B}T\ln2=20$ meV with $\frac{\hbar \;\cdot \;2\pi /\lambda }{k_{B}T\ln2}\gg 1$, so that information can be recorded at physiological temperatures with no risk of thermal degradation. If the Landauer principle is applied for holographic information processing, the maximum wavelength for information processing will be 60 μm. We will focus on photons with shorter wavelengths than this value to investigate holographic information processing.

A control theory was developed based on morphological computation using input–output equations in [62]. Adopting multiple layers in a hierarchy as a model of neocortex covered by celebrospinal fluid, dura and skull, we have checked how target photon fields $A_{i}^{\left(N\right)}$ inducing step-function-like distributions of charged Bose fields $|{\bar{\varphi }}^{\left(N\right)}{|}^{2}$ are achieved by external photons fields. In numerical simulations, we adopt damping input functions $A_{i}^{\left(0\right)}$ to achieve step-function-like distributions of charged bosons by factor $\gamma_{2}$ in Equations (A90) and (A91). We explain the reason why we adopt damping input functions as follows. Our control theory corresponds to manipulation of the density distributions ρ in the equation of continuity in hydrodynamics,

(12) $$\begin{array}{c} \frac{\partial }{\partial x^{0}}\rho \left(x\right)=\partial_{i}\left(\rho \left(x\right)V^{i}\left(x\right)\right), \end{array}$$

by the velocity distributions $V^{i}\left(x\right)$. What we can manipulate is the velocity corresponding to the vector potential $A_{i}-\frac{\partial_{i}\beta }{e}$ multiplled by $\frac{e}{m}$ in Equation (A69). Hence, the velocity is nonzero at later times $mx^{0}∼200$ in Section 4, charged bosons will collect into the peripheral regions around $|x^{1}|∼N_{s}a_{s}$ and $|x^{2}|∼N_{s}a_{s}$. Using damping input functions, density distributions of charged bosons for holograms stops evolving over time, and target binary holograms are achieved. We find slight distortions in the step functions in Figure 6f at $mx^{0}=200$. Although distortions originate from the Laplacian of $\left(\partial_{i}^{2}\right)\left|{\bar{\varphi }}^{\left(N\right)}\right|$ in $A^{0}-\partial_{0}\beta /e$ in Equation (A87) can gradually become comparable to damping $A_{i}-\partial_{i}\beta /e$ over time elapsed, they do not seriously affect the step functions of binary holograms in present numerical simulations.

We also need to consider geometry to develop a proper control theory. In this work, we have investigated a 2-dimensional flat surface with multiple layers. We then proposed non-invasive manipulation of holograms by external electromagnetic fields penetrating thorough multiple layers. However, we encounter various structures of holograms for information storage by water molecular conformational states around spherical, toroidal and cylindrical forms for neurons, glia cells, microfilaments and microtubules. To manipulate holograms with various forms of water states, we have to adopt a hemispherical or spherical surface headset covering our head to achieve target functions of electromagnetic fields and holograms composed of water molecular conformational states around 3-dimensional cells and the cytoskeleton.

Once our subjective experiences and memory are manipulated by external stimuli, we find one-to-one correspondence between holograms induced by external input functions and our subjective experiences. Our approach might indicate the reductionism of subjective experiences to holograms of water media.

## 6. Concluding Remarks and Perspectives

Vision is perhaps the most important link between the outside world of the observable phenomena and the inner life of our mind. We more or less know how it works based on the photoreceptors of the eye’s retina and the subsequent optic nerve activation. But where is the image of the outside world formed inside the brain? Ruppert Sheldrake hypothesized that we actually send waves outside our body to probe the space around us. On the other hand, Karl Pribram proposed a holographic image formation within our brains. Which of these ideas is closer to the truth? We argue that the holographic brain hypothesis has merits that have not yet been fully explored. One of the possible benefits would be simultaneous integration and synchronization of sensory inputs into a coherent whole.

Karl Pribram’s holonomic brain theory (quantum holography) invoked quantum mechanics to explain higher-order processing by the mind. He argued that his holonomic model solved the binding problem. Pribram collaborated with the famous physicist David Bohm in his work on quantum approaches to the functioning of the mind [78]. He proposed that ordered water at dendritic membrane surfaces undergoes Bose–Einstein condensation forming a large-scale coherent quantum state, which would support ideas such as quantum brain dynamics proposed earlier by Umezawa and collaborators. This, somewhat far-fetched prediction received a boost after decades of silence. In 2022, neuroscientists [39] reported experimental MRI results obtained from human participants, which imply the involvement of nuclear proton spins of brain water molecules in an entangled quantum state. This is a major step toward providing evidence that at least some brain functions have hallmarks of non-classical, quantum behavior and are involved in consciousness.

We have investigated Quantum Electrodynamics with non-relativistic charged bosons (a model of water molecular conformational states) corresponding to holographic brain theory by Pribram. A super-radiance solution in holographic brain theory is derived, and holographic aspect has been shown with deriving the limitation of memory capacity of a neocortex. We can propose a control theory of holograms by adopting morphological computation. We have shown how binary holograms are manipulated by external photon fields in a hierarchical model with multiple layers (input layer: scalp, intermediate layers: skull, dura, and celebrospinal fluid, and output layer: neocortex). We intend to apply our approach to manipulate holographic memory and subjective experiences by external electromagnetic fields in a future experimental study.

## Appendix A. Derivation of Super-Radiance Solution

We shall derive a super-radiance solution via a microtubule.

The Lagrangian density for QED without gauge fixing is,

(A1) $$\begin{array}{c} L=-\frac{1}{4}F^{\mu \nu }\left[A\right]F_{\mu \nu }\left[A\right]+\varphi^{*}\left[i\frac{\partial }{\partial x^{0}}+eA^{0}+\frac{{\left(\nabla_{i}-ieA_{i}\right)}^{2}}{2m}-U\left(x\right)\right]\varphi , \end{array}$$

with electromagnetic field tensor $F^{\mu \nu }\left[A\right]=\partial^{\mu }A^{\nu }-\partial^{\nu }A^{\mu }$, scalar potential $A^{0}$, vector potential $A^{i}$ (i=1,2,3), charged Bose fields φ and its complex conjugate $\varphi^{*}$, potential energy U(x), mass *m* and effective charge *e* for ionized water degrees of freedom.

We shall adopt the Coulomb gauge, namely $A^{0}=0$ and $\nabla_{i}A_{i}=0$. Time-evolution equations are then derived by the Euler–Lagrangian equations for Lagrangian given in Equation (A1) as,

(A2) $$\begin{array}{c} \left(i\frac{\partial }{\partial x^{0}}+\frac{\nabla_{i}^{2}}{2m}-U\left(x\right)-\frac{e^{2}A_{i}^{2}}{2m}-\frac{ieA_{i}\nabla_{i}}{m}\right)\varphi \left(x\right)=0, \end{array}$$

with its complex conjugate, and,

(A3) $$\begin{array}{c} \partial^{\nu }F_{\nu i}=J_{i}, \end{array}$$

with,

(A4) $$\begin{array}{c} J_{i}=-\frac{ie}{2m}\left[\varphi^{*}\left(\nabla_{i}-ieA_{i}\right)\varphi -\left(\left(\nabla_{i}+ieA_{i}\right)\varphi^{*}\right)\varphi \right]. \end{array}$$

We call Equation (A2) Schrödinger-like equation in the framework of QFT with modeling dynamics of water molecular conformational states. We also use the Klein–Gordon equation in Equation (A3). The Schrödinger-like Equation (A2) represents the extension of neural-wave equation adopted by Pribram [13] with involving the coupling with photon fields. Using the Schrödinger-like Equation (A2) and its complex conjugate, we can derive the charge conservation law as represented by,

(A5) $$\begin{array}{ccc} \partial^{0}J_{0} & = & -\partial^{i}J_{i}, \end{array}$$

(A6) $$\begin{array}{ccc} J_{0} & \equiv & -e\varphi^{*}\varphi . \end{array}$$

Using the above equation and Equation (A3), we can derive,

(A7) $$\begin{array}{c} \partial^{0}J_{0}=-\partial^{i}J_{i}=-\partial^{i}\partial^{\nu }F_{\nu i}=\partial^{\nu }\partial^{\mu }F_{\nu \mu }-\partial^{i}\partial^{\nu }F_{\nu i}=\partial^{0}\partial^{\nu }F_{\nu 0},\\ \mathrm{or},\;\;\partial^{\nu }F_{\nu 0}=J_{0}, \end{array}$$

where time-independent terms that might represent boundary conditions are set to be zero.

Next we shall expand φ by eigenfunctions $b_{n}\left(x^{0}\right)\phi_{n}\left(x\right)$ for conformational energy $E_{n}$ (for vibrational motion, and so on) as,

(A8) $$\begin{array}{ccc} \varphi (x) & = & \sum_{n}b_{n}\left(x^{0}\right)\phi_{n}\left(x\right), \end{array}$$

(A9) $$\begin{array}{ccc} \left(-\frac{\nabla_{i}^{2}}{2m}+U\left(x\right)+\frac{e^{2}A_{i}^{2}}{2m}\right)\phi_{n} & = & E_{n}\phi_{n}. \end{array}$$

Then normalization condition is expressed as $\int_{x}{\left|\varphi \left(x\right)\right|}^{2}=\sum_{n}{\left|b_{n}\left(x^{0}\right)\right|}^{2}=N$ with the number of water molecules *N*.

We consider vibrational motion in $x^{3}$ direction and set,

(A10) $$\begin{array}{c} |\nabla_{3}\phi_{n}\left|\gg \right|\nabla_{1}\phi_{n}\left|,\;\right|\nabla_{2}\phi_{n}|. \end{array}$$

We then adopt $A_{1}=A_{2}=0$. Time-evolution equation for $A_{3}$ is derived from Equation (A3) as,

(A11) $$\begin{array}{c} \left(\partial_{0}^{2}-\nabla_{1}^{2}-\nabla_{2}^{2}\right)A_{3}+\frac{e^{2}{\left|\varphi \right|}^{2}}{m}A_{3}=-\frac{ie}{2m}\left(\varphi^{*}\nabla_{3}\varphi -\varphi \nabla_{3}\varphi^{*}\right). \end{array}$$

We adopt two-energy-level approximation for charged Bose field φ(x) with n=0,1 and set,

(A12) $$\begin{array}{c} b_{n}\left(x^{0}\right)=e^{-iE_{n}x^{0}}\chi_{n}\left(x^{0}\right), \end{array}$$

where $E_{0}$ and $E_{1}$ represent energy eigenvalues in molecular conformational states for the ground state and 1st excited state, respectively. The energy difference $\Omega \equiv E_{1}-E_{0}$ corresponds to a mode of absorption in water spectroscopy. We find $\sum_{n=0,1}{\left|\chi_{n}\left(x^{0}\right)\right|}^{2}=N$, the number of water molecules in particular conformational states.

We next expand vector potential $A_{3}\left(x\right)$ by,

(A13) $$\begin{array}{c} A_{3}\left(x\right)=\int_{k^{1},k^{2}}\frac{1}{2}\left[\alpha_{3,k}\left(x\right)e^{-i(\omega_{k}x^{0}-k\;\cdot \;x)}+\alpha_{3,k}^{*}\left(x\right)e^{i(\omega_{k}x^{0}-k\;\cdot \;x)}\right], \end{array}$$

with integration of two-dimensional momenta $k=(k^{1},k^{2},0)$, namely $\int_{k}=\int \frac{dk^{1}}{2\pi }\frac{dk^{2}}{2\pi }$. Here we set $\omega_{k}=\sqrt{{\left(k^{1}\right)}^{2}+{\left(k^{2}\right)}^{2}+\frac{e^{2}{\left|\bar{\varphi }\right|}^{2}}{m}}$ with spatial average $|\bar{\varphi }{|}^{2}=\frac{N}{V}$ with the 3-dimensional volume $V=L_{1}L_{2}L_{3}$ (with size $L_{i}$, i=1,2,3). Here the bar represents the spatial average in this section.

Using relations (A12) and (A13), definition $\Omega \equiv E_{1}-E_{0}$, and integration with $\int_{x}\phi_{0,1}^{*}\left(x\right)\times$, the Schrödinger-like Equation (A2) includes time-evolution equations for modes $\chi_{0}$ and $\chi_{1}$ as,

(A14) $$\begin{array}{ccc} i\frac{\partial \chi_{0}}{\partial x^{0}} & = & \frac{e}{m}\int_{x}\int_{k^{1},k^{2}}[\frac{1}{2}\left[\alpha_{3,k}\left(x\right)e^{-i(\omega_{k}x^{0}-k\;\cdot \;x)}+\alpha_{3,k}^{*}\left(x\right)e^{i(\omega_{k}x^{0}-k\;\cdot \;x)}\right]\\ & & \times \left[e^{-i\Omega x^{0}}\left(\phi_{0}^{*}i\nabla_{3}\phi_{1}\right)\chi_{1}+\left(\phi_{0}^{*}i\nabla_{3}\phi_{0}\right)\chi_{0}\right], \end{array}$$

(A15) $$\begin{array}{ccc} i\frac{\partial \chi_{1}}{\partial x^{0}} & = & \frac{e}{m}\int_{x}\int_{k^{1},k^{2}}[\frac{1}{2}\left[\alpha_{3,k}\left(x\right)e^{-i(\omega_{k}x^{0}-k\;\cdot \;x)}+\alpha_{3,k}^{*}\left(x\right)e^{i(\omega_{k}x^{0}-k\;\cdot \;x)}\right]\\ & & \times \left[e^{i\Omega x^{0}}\left(\phi_{1}^{*}i\nabla_{3}\phi_{0}\right)\chi_{0}+\left(\phi_{1}^{*}i\nabla_{3}\phi_{1}\right)\chi_{1}\right]. \end{array}$$

We adopt the rotating-wave approximation where we leave only resonant mode $\Omega =\omega_{k}$ and neglect non-resonant modes. The above equations are then rewritten by,

(A16) $$\begin{array}{ccc} i\frac{\partial \chi_{0}}{\partial x^{0}} & = & \frac{e}{2m}\int_{k^{1},k^{2};\omega_{k}=\Omega }\alpha_{3,k}^{*}\int_{x}e^{-ik\;\cdot \;x}\left(\phi_{0}^{*}i\nabla_{3}\phi_{1}\right)\chi_{1}, \end{array}$$

(A17) $$\begin{array}{ccc} i\frac{\partial \chi_{1}}{\partial x^{0}} & = & \frac{e}{2m}\int_{k^{1},k^{2};\omega_{k}=\Omega }\alpha_{3,k}\int_{x}e^{ik\;\cdot \;x}\left(\phi_{1}^{*}i\nabla_{3}\phi_{0}\right)\chi_{0}. \end{array}$$

Next we use the relation,

(A18) $$\begin{array}{ccc} \frac{\partial^{2}}{\partial {\left(x^{0}\right)}^{2}}A_{3} & = & \int_{k^{1},k^{2}}e^{-i(\omega_{k}x^{0}-k\;\cdot \;x)}\left(-\omega_{k}^{2}-2i\omega_{k}\frac{\partial }{\partial x^{0}}+\frac{\partial^{2}}{\partial {\left(x^{0}\right)}^{2}}\right)\frac{1}{2}\alpha_{3,k}+\cdots . \end{array}$$

the Fourier transformation with momenta $l=(l^{1},l^{2},0)$ of $\frac{e^{2}{\left|\varphi \right|}^{2}}{m}A_{3}$ expanded by,

(A19) $$\begin{array}{ccc} \int_{x}e^{-il\;\cdot \;x}\frac{e^{2}{\left|\varphi \left(x\right)\right|}^{2}}{m}A_{3} & = & \int_{x}e^{-il\;\cdot \;x}\frac{e^{2}{\left|\varphi \left(x\right)\right|}^{2}}{m}\int_{k^{1},k^{2}}\left[\frac{1}{2}\alpha_{3,k}e^{-i(\omega_{k}x^{0}-k\;\cdot \;x)}+c.c.\right]\\ & & \left(k∼l\;\mathrm{dominant}\right)\\ & ∼ & \frac{e^{2}}{m}\int_{x}{\left|\varphi \left(x\right)\right|}^{2}\frac{1}{2}\alpha_{3,l}e^{-i\omega_{l}x^{0}}\int_{k^{1},k^{2};k=l}+\left(\mathrm{term}\;\mathrm{with}\;\alpha_{3,-l}^{*}\right)\\ & = & \frac{e^{2}{\left|\bar{\varphi }\right|}^{2}}{m}L_{1}L_{2}L_{3}\frac{1}{2}\alpha_{3,l}\frac{1}{L_{1}L_{2}}e^{-i\omega_{l}x^{0}}+\cdots \\ & = & \frac{e^{2}{\left|\bar{\varphi }\right|}^{2}}{m}L_{3}\times \frac{1}{2}\alpha_{3,l}e^{-i\omega_{l}x^{0}}+\cdots , \end{array}$$

with $\int_{k^{1},k^{2};k=l}=\frac{1}{L_{1}L_{2}}\sum_{k^{1}k^{2};k=l}=\frac{1}{L_{1}L_{2}}$, the relation

(A20) $$\begin{array}{c} \left(-\omega_{l}^{2}+{\left(l^{1}\right)}^{2}+{\left(l^{2}\right)}^{2}+\frac{e^{2}{\left|\bar{\varphi }\right|}^{2}}{m}\right)L_{3}e^{-i\omega_{l}x^{0}}=0, \end{array}$$

due to the relation $\omega_{l}=\sqrt{{\left(l^{1}\right)}^{2}+{\left(l^{2}\right)}^{2}+\frac{e^{2}{\left|\bar{\varphi }\right|}^{2}}{m}}$, the conditions,

(A21) $$\begin{array}{c} \left|\frac{\partial \alpha_{3,l}}{\partial x^{0}}\right|\ll \omega_{l}|\alpha_{3,l}|,\;\;\;\;\left|\frac{\partial \alpha_{3,l}}{\partial x^{i}}\right|\ll \left|l^{i}\alpha_{3,l}\right|. \end{array}$$

We then rewrite the Fourier transformation in Equation (A11) as,

(A22) $$\begin{array}{ccc} & & \left(-i\omega_{l}\frac{\partial }{\partial x^{0}}-il^{1}\frac{\partial }{\partial x^{1}}-il^{2}\frac{\partial }{\partial x^{2}}\right)\alpha_{3,l}e^{-i\omega_{l}x^{0}}L_{3}\\ & = & -\frac{ie}{2m}\int_{x}e^{-il\;\cdot \;x}[e^{i\Omega x^{0}}\chi_{1}^{*}\chi_{0}\left(\phi_{1}^{*}\nabla_{3}\phi_{0}\right)+e^{-i\Omega x^{0}}\chi_{0}^{*}\chi_{1}\left(\phi_{0}^{*}\nabla_{3}\phi_{1}\right)\\ & & -e^{-i\Omega x^{0}}\chi_{0}^{*}\chi_{1}\left(\phi_{1}\nabla_{3}\phi_{0}^{*}\right)-e^{i\Omega x^{0}}\chi_{0}\chi_{1}^{*}\left(\phi_{0}\nabla_{3}\phi_{1}^{*}\right)]. \end{array}$$

Taking the resonant mode $\omega_{l}=\Omega$ in rotating-wave approximation and replace l by k in notation, we arrive at,

(A23) $$\begin{array}{ccc} \left[\frac{\partial \alpha_{3,k}}{\partial x^{0}}+\frac{k^{1}}{\omega_{k}}\frac{\partial \alpha_{3,k}}{\partial x^{1}}++\frac{k^{2}}{\omega_{k}}\frac{\partial \alpha_{3,k}}{\partial x^{2}}\right] & = & -i\frac{e}{\omega_{k}L_{3}}\chi_{0}^{*}\chi_{1}\int_{x}e^{-ik\;\cdot \;x}\phi_{0}^{*}\frac{i\nabla_{3}}{m}\phi_{1}. \end{array}$$

Next we use the dipole approximation $e^{ik\;\cdot \;x}≃1$ where the system size is sufficiently small compared with the wavelength of photons. Then Equations (A16), (A17) and (A23) are rewritten by,

(A24) $$\begin{array}{ccc} i\frac{\partial \chi_{0}}{\partial x^{0}} & = & \frac{e}{2}\int_{k^{1},k^{2};\omega_{k}=\Omega }\alpha_{3,k}^{*}\int_{x}\left(\phi_{0}^{*}\frac{i\nabla_{3}}{m}\phi_{1}\right)\chi_{1}, \end{array}$$

(A25) $$\begin{array}{ccc} i\frac{\partial \chi_{1}}{\partial x^{0}} & = & \frac{e}{2}\int_{k^{1},k^{2};\omega_{k}=\Omega }\alpha_{3,k}\int_{x}\left(\phi_{1}^{*}\frac{i\nabla_{3}}{m}\phi_{0}\right)\chi_{0}, \end{array}$$

and,

(A26) $$\begin{array}{ccc} \left[\frac{\partial \alpha_{3,k}}{\partial x^{0}}+\frac{k^{1}}{\omega_{k}}\frac{\partial \alpha_{3,k}}{\partial x^{1}}++\frac{k^{2}}{\omega_{k}}\frac{\partial \alpha_{3,k}}{\partial x^{2}}\right] & = & -i\frac{e}{\omega_{k}L_{3}}\chi_{0}^{*}\chi_{1}\int_{x}\phi_{0}^{*}\frac{i\nabla_{3}}{m}\phi_{1}. \end{array}$$

We shall define,

(A27) $$\begin{array}{c} J_{n_{1}n_{2}}\equiv \int_{x}\phi_{n_{1}}^{*}\frac{i\nabla_{3}}{m}\phi_{n_{2}}, \end{array}$$

with $n_{1},n_{2}=0,1$ and define,

(A28) $$\begin{array}{c} A\equiv \int_{k^{1},k^{2};\omega_{k}=\Omega }\alpha_{3,k}. \end{array}$$

We next adopt the relation,

(A29) $$\begin{array}{ccc} \int_{k^{1},k^{2};\omega_{k}=\Omega } & = & 2\pi \Omega^{2}\int wdw\delta \left(w^{2}-\frac{\Omega^{2}-\frac{e^{2}{\left|\bar{\varphi }\right|}^{2}}{m}}{\Omega^{2}}\right)\frac{1}{{\left(2\pi \right)}^{2}}\\ & = & \frac{\Omega^{2}}{4\pi }, \end{array}$$

where we have used $w=\frac{\sqrt{{\left(k^{1}\right)}^{2}+{\left(k^{2}\right)}^{2}}}{\Omega }$ and considered the case when $\Omega \geq \frac{e^{2}{\left|\bar{\varphi }\right|}^{2}}{m}$ is satisfied. Using Equations (A27)–(A29), time-evolution Equations (A24)–(A26) can be simplified to,

(A30) $$\begin{array}{ccc} \frac{\partial \chi_{0}}{\partial x^{0}} & = & -i\frac{e}{2}J_{01}\chi_{1}A^{*}, \end{array}$$

(A31) $$\begin{array}{ccc} \frac{\partial \chi_{1}}{\partial x^{0}} & = & -i\frac{e}{2}J_{10}\chi_{1}A, \end{array}$$

(A32) $$\begin{array}{ccc} \frac{\partial A}{\partial x^{0}} & = & -\frac{ie\Omega }{4\pi L_{3}}J_{01}\chi_{0}^{*}\chi_{1}. \end{array}$$

We shall consider the real $J_{01}=J_{10}$, set $g\equiv \frac{eJ_{10}}{2}$. We also replace A→−iA and assume real A. The above equations are then rewritten by,

(A33) $$\begin{array}{ccc} \frac{\partial \chi_{0}}{\partial x^{0}} & = & gA\chi_{1}, \end{array}$$

(A34) $$\begin{array}{ccc} \frac{\partial \chi_{1}}{\partial x^{0}} & = & -gA\chi_{0}, \end{array}$$

(A35) $$\begin{array}{ccc} \frac{\partial A}{\partial x^{0}} & = & \frac{g\Omega }{4\pi L_{3}}\times \left(2\chi_{0}^{*}\chi_{1}\right). \end{array}$$

We find the number conservation law of water molecules $\partial_{0}\left(\right|\chi_{0}{|}^{2}+|\chi_{1}{|}^{2})=0$ from above equation.

We shall define $Z\left(x^{0}\right)\equiv |\chi_{1}{|}^{2}-{\left|\chi_{0}\right|}^{2}$ and $R\left(x^{0}\right)\equiv 2\chi_{0}^{*}\chi_{1}$. Using Equations (A33)–(A35), the *Z* and *R* are found to satisfy,

(A36) $$\begin{array}{ccc} \frac{\partial Z}{\partial x^{0}} & = & -2gAR, \end{array}$$

(A37) $$\begin{array}{ccc} \frac{\partial R}{\partial x^{0}} & = & 2gAZ, \end{array}$$

(A38) $$\begin{array}{ccc} \frac{\partial A}{\partial x^{0}} & = & \frac{g\Omega }{4\pi L_{3}}R. \end{array}$$

The above equations correspond to the Maxwell–Bloch equations in Quantum Electrodynamics [36,68,79]. We can derive the energy conservation law represented by,

(A39) $$\begin{array}{c} \frac{\partial }{\partial x^{0}}\left[\frac{1}{2}A^{2}+\frac{\Omega }{8\pi L_{3}}Z\right]=0, \end{array}$$

and the number conservation law $\partial_{0}\left(Z^{2}+R^{2}\right)=0$ or $Z^{2}+R^{2}=N^{2}$.

We shall introduce $\theta (x^{0})$ with Z=Ncosθ, and R=Nsinθ. Substituting R=Nsinθ into Equation (A37), we find,

(A40) $$\begin{array}{c} \frac{\partial \theta }{\partial x^{0}}=2gA. \end{array}$$

Hence we find the relation

(A41) $$\begin{array}{c} \theta \left(x^{0}\right)=\theta_{0}+2g\int_{t_{0}}^{x^{0}}A. \end{array}$$

Setting the length $L_{3}$ for release of radiation, Equation (A38) is rewritten by,

(A42) $$\begin{array}{ccc} \frac{\partial A}{\partial x^{0}}+\frac{1}{L_{3}}A & = & \frac{g\Omega }{4\pi L_{3}}N\sin\theta . \end{array}$$

When we consider the case $\partial_{0}\ll \frac{1}{L_{3}}$, we find the relation,

(A43) $$\begin{array}{c} \frac{\partial \theta }{\partial x^{0}}=\frac{g^{2}\Omega N}{2\pi }\sin\theta . \end{array}$$

The solution of the above equation is,

(A44) $$\begin{array}{c} \theta =2\tan^{-1}\left(\exp\left(\frac{g^{2}\Omega N}{2\pi }x^{0}\right)\tan\frac{\theta_{0}}{2}\right). \end{array}$$

We then find,

(A45) $$\begin{array}{ccc} A & = & \frac{1}{2g}\frac{\partial \theta }{\partial x^{0}}\\ & = & \frac{g\Omega N}{4\pi }{\left[\cosh\left(\frac{x^{0}-\tau_{0}}{\tau_{R}}\right)\right]}^{-1}, \end{array}$$

with,

(A46) $$\begin{array}{c} \tau_{R}=\frac{2\pi }{g^{2}\Omega N}, \end{array}$$

and $\tau_{0}=-\tau_{R}\ln\tan\frac{\theta_{0}}{2}$. Then the amplitude of electric field $E_{3}$ is,

(A47) $$\begin{array}{c} E_{3}∼\Omega A=\frac{g\Omega^{2}N}{4\pi }{\left[\cosh\left(\frac{x^{0}-\tau_{0}}{\tau_{R}}\right)\right]}^{-1}, \end{array}$$

with the necessary condition $\Omega \geq \frac{e^{2}{\left|\bar{\varphi }\right|}^{2}}{m}$.

## Appendix B. Holographic Aspect

We introduce holographic aspect in QED. The Klein–Gordon Equation (A3) in the Coulomb gauge in Appendix A is rewritten by,

(A48) $$\begin{array}{c} \left[{\left(\partial_{0}\right)}^{2}-{\left(\partial_{j}\right)}^{2}+\frac{e^{2}{\left|\varphi \right|}^{2}}{m}+M^{2}\right]A_{i}=M^{2}{\tilde{A}}_{i}+O\left(\partial \varphi ,\partial \varphi^{*}\right), \end{array}$$

where we have added the term $M^{2}A_{i}$ for the contribution to Meissner effect with parameter $M^{2}$ by ionic bioplasma in biological systems and the term with external input field ${\tilde{A}}_{i}=be^{i(k^{0}x^{0}-k^{j}x^{j})}$ as coherent super-radiant waves. The special solution of the above equation is,

(A49) $$\begin{array}{ccc} A_{i} & = & \frac{M^{2}be^{i(k^{0}x^{0}-k^{j}x^{j})}}{M^{2}+\frac{e^{2}{\left|\varphi \right|}^{2}}{m}}+O\left(\partial \varphi ,\partial \varphi^{*}\right)\\ & = & \left(1-\frac{e^{2}{\left|\varphi \right|}^{2}}{M^{2}m}+\cdots \right)be^{i(k^{0}x^{0}-k^{j}x^{j})}+\cdots . \end{array}$$

We shall consider holographic memories are recorded on 2-dimensional surface in $x^{1}$ and $x^{2}$ directions (with thickness for additional 1-dimensional direction). We set,

(A50) $$\begin{array}{c} {\tilde{A}}_{i}=r\left(x^{1},x^{2}\right), \end{array}$$

with reference wave *r* on the 2-dimensional surface. We shall then consider the case ${\left|\varphi \right|}^{2}$ is a function of interference patterns of reference wave $r(x^{1},x^{2})$ and object wave $o(x^{1},x^{2})$ given by,

(A51) $$\begin{array}{c} {\left|\varphi \right|}^{2}\propto {\left|r\left(x^{1},x^{2}\right)+o\left(x^{1},x^{2}\right)\right|}^{2}. \end{array}$$

Then the special solution in Equation (A49) can be rewritten by,

(A52) $$\begin{array}{c} {\tilde{A}}_{i}\propto {r(|r|}^{2}+{\left|o\right|}^{2}+r^{*}o+ro^{*})+\left(\mathrm{other}\;\mathrm{terms}\right). \end{array}$$

The above solution involves the term $|r^{2}|o$ for image reconstruction in holography. When super-radiant wave is imposed on 2-dimensional holograms, image *o* is reconstructed.

## Appendix C. Time-Evolution Equations in Control Theory

We shall derive time-evolution equations in the control theory in QED.

We introduce the Lagrangian density in QED with non-relativistic charged bosons corresponding to holographic brain theory by Pribram [13], derive time-evolution equations, and extend the theory to that in a hierarchy.

We begin with the Lagrangian density in background field gauge [80,81,82,83] given by,

(A53) $$\begin{array}{ccc} L(x) & = & -\frac{1}{4}F_{\mu \nu }\left[A+a\right]F^{\mu \nu }\left[A+a\right]-\frac{1}{2\xi }{\left(\partial_{\mu }a^{\mu }\right)}^{2}\\ & & +\varphi^{*}\left(i\frac{\partial }{\partial x^{0}}+e\left(A^{0}+a^{0}\right)+\frac{{\left(\nabla_{i}-ie\left(A_{i}+a_{i}\right)\right)}^{2}}{2m}\right)\varphi , \end{array}$$

with electromagnetic field tensor $F_{\mu \nu }\left[A\right]=\partial_{\mu }A_{\nu }-\partial_{\nu }A_{\mu }$ involving background field $A_{\mu }\left(x\right)$ and its quantum fluctuation $a_{\mu }\left(x\right)$, gauge fixing parameter ξ, non-relativistic charged boson fields $\varphi^{\left(*\right)}\left(x\right)$, elementary charge *e* and mass of bosons *m*. The above Lagrangian is invariant under the type-I gauge transformation written by,

(A54) $$\begin{array}{c} \varphi \left(x\right)\to e^{i\alpha (x)}\varphi \left(x\right),\;\;\;\varphi^{*}\left(x\right)\to e^{-i\alpha (x)}\varphi^{*}\left(x\right),\;\;\;A_{\mu }\left(x\right)\to A_{\mu }\left(x\right)+\frac{1}{e}\partial_{\mu }\alpha \left(x\right),\;\;\;a_{\mu }\left(x\right)\to a_{\mu }\left(x\right). \end{array}$$

We shall set the gauge-fixing condition for quantum fluctuation $a_{\mu }$ as,

(A55) $$\begin{array}{c} a^{0}=0,\;\;\;\xi =1. \end{array}$$

Using the Lagrangian density in Equation (A53) and adopting 2-Particle-Irreducible (2PI) Effective Action Technique [84,85,86] in the closed-time path formalism [87,88], we can derive the following 2PI effective action,

(A56) $$\begin{array}{ccc} \Gamma_{2\mathrm{PI}}\left[A,\bar{a},\bar{\varphi },{\bar{\varphi }}^{*},\Delta ,D\right] & = & \int_{C}d^{4}x[-\frac{1}{4}F_{\mu \nu }\left[A+\bar{a}\right]F^{\mu \nu }\left[A+\bar{a}\right]-\frac{{\left(\partial^{i}{\bar{a}}_{i}\right)}^{2}}{2}\\ & & +{\bar{\varphi }}^{*}\left(i\frac{\partial }{\partial x^{0}}+eA^{0}+\frac{{\left(\nabla_{i}-ie\left(A_{i}+{\bar{a}}_{i}\right)\right)}^{2}}{2m}\right)\bar{\varphi }]\\ & & +\frac{i}{2}\mathrm{Tr}\ln D^{-1}+\frac{1}{2}\mathrm{Tr}\left(iD_{0}^{-1}D\right)+i\mathrm{Tr}\ln\Delta^{-1}+\mathrm{Tr}\left(i\Delta_{0}\Delta \right)\\ & & +\frac{1}{2}\Gamma_{2}\left[A+\bar{a},\bar{\varphi },{\bar{\varphi }}^{*},\Delta ,D\right], \end{array}$$

where bar represents the expectation values $\bar{a}=\langle a\rangle =\mathrm{Tr}\left(\rho a\right)$ and ${\bar{\varphi }}^{\left(*\right)}=\langle \varphi^{\left(*\right)}\rangle =\mathrm{Tr}\left(\rho \varphi^{\left(*\right)}\right)$ with density matrix ρ, and C represents the closed-time path involving contour 1 from −∞ to *∞* and contour 2 from *∞* to −∞. In the 3rd term, $i\Delta_{0}^{-1}$ represents,

(A57) $$\begin{array}{ccc} i\Delta_{0}^{-1}\left(x,y\right) & = & \frac{\delta^{2}\int_{w}L\left(w\right)}{\delta \varphi^{*}\left(x\right)\delta \varphi \left(y\right)}\\ & = & \left(i\frac{\partial }{\partial x^{0}}+eA^{0}+\frac{{\left(\nabla_{i}-ie\left(A_{i}+{\bar{a}}_{i}\right)\right)}^{2}}{2m}\right)\delta_{C}\left(x-y\right). \end{array}$$

In the 5th term in Equation (A56), $iD_{0}^{-1}$ represents,

(A58) $$\begin{array}{ccc} iD_{0,ij}^{-1}\left(x,y\right) & = & \frac{\delta^{2}\int_{w}L\left(w\right)}{\delta a^{i}\left(x\right)\delta a^{j}\left(y\right)}\\ & = & -\left(\partial_{x}^{2}+\frac{e^{2}{\left|\bar{\varphi }\left(x\right)\right|}^{2}}{m}\right)\delta_{ij}\delta_{C}\left(x-y\right), \end{array}$$

with subscripts for spatial cordinates i,j=1,2,3. The *D* in the 2nd and 3rd terms in Equation (A56) represents the Green’s functions for for photon fields given by,

(A59) $$\begin{array}{c} D_{ij}\left(x,y\right)=\langle T_{C}\left(a_{i}\left(x\right)a_{j}\left(y\right)\right)\rangle . \end{array}$$

The Δ in the 4th and the 5th terms in Equation (A56) represents the Green’s function for charged Bose field given by,

(A60) $$\begin{array}{c} \Delta \left(x,y\right)=\langle T_{C}\left(\delta \varphi \left(x\right)\delta \varphi^{*}\left(y\right)\right)\rangle , \end{array}$$

with quantum fluctuations $\delta \varphi^{\left(*\right)}=\varphi^{\left(*\right)}-{\bar{\varphi }}^{\left(*\right)}$. It is possible to express Δ by 2×2 matrix notation in the closed-time path as,

(A61) $$\begin{array}{c} \Delta \left(x,y\right)=\left[\begin{array}{cc} \Delta^{11}\left(x,y\right) & \Delta^{12}\left(x,y\right)\\ \Delta^{21}\left(x,y\right) & \Delta^{22}\left(x,y\right) \end{array}\right]=\left[\begin{array}{cc} \langle T\delta \varphi \left(x\right)\delta \varphi^{*}\left(y\right)\rangle & \langle \delta \varphi^{*}\left(y\right)\delta \varphi_{(}x)\rangle \\ \langle \delta \varphi \left(x\right)\delta \varphi^{*}\left(y\right)\rangle & \langle \tilde{T}\delta \varphi \left(x\right)\delta \varphi^{*}\left(y\right)\rangle \end{array}\right], \end{array}$$

with time-ordered product T and anti-time-ordered product $\tilde{T}$. The $\Gamma_{2}$ in Equation (A56) represents all the 2PI diagrams [89].

Differentiating 2PI effective action by fields *A* (or $\bar{a}$), ${\bar{\varphi }}^{\left(*\right)}$ and Green’s functions *D* and Δ and setting ${\bar{a}}_{i}=0$, we can derive time-evolution equations. The equation $\frac{\delta \Gamma_{2\mathrm{PI}}}{\delta {\bar{a}}^{i}}{|}_{{\bar{a}}_{i}=0}=\frac{\delta \Gamma_{2\mathrm{PI}}}{\delta A^{i}}{|}_{{\bar{a}}_{i}=0}=0$ gives the following equation,

(A62) $$\begin{array}{ccc} \partial^{\nu }F_{\nu i} & = & -\frac{ie}{2m}\left[{\bar{\varphi }}^{*}\left(\nabla_{i}-ieA_{i}\right)\bar{\varphi }-\left(\left(\nabla_{i}+ieA_{i}\right){\bar{\varphi }}^{*}\right)\bar{\varphi }\right]-\frac{1}{2}\frac{\delta \Gamma_{2}}{\delta A^{i}}\\ & & -\frac{ie}{2m}\left[\left(\nabla_{x_{1},i}-ieA_{i}\left(x_{1}\right)\right)\Delta^{11}\left(x_{1},x\right){|}_{x_{1}=x}-\left(\nabla_{x_{2},i}+ieA_{i}\left(x_{2}\right)\right)\Delta^{11}\left(x,x_{2}\right){|}_{x_{2}=x}\right]. \end{array}$$

Using the relation $\frac{\delta \Gamma_{2\mathrm{PI}}}{\delta {\bar{\varphi }}^{*}}{|}_{{\bar{a}}_{i}=0}=0$, we can derive the Schrödinger-like equation,

(A63) $$\begin{array}{c} \left[i\frac{\partial }{\partial x^{0}}+eA_{0}+\frac{{\left(\nabla_{i}-ieA_{i}\right)}^{2}}{2m}-\frac{e^{2}D_{ii}^{11}\left(x,x\right)}{2m}\right]\bar{\varphi }\left(x\right)+\frac{1}{2}\frac{\delta \Gamma_{2}}{\delta {\bar{\varphi }}^{*}}=0. \end{array}$$

Using the relation $\frac{\delta \Gamma_{2\mathrm{PI}}}{\delta \bar{\varphi }}{|}_{{\bar{a}}_{i}=0}=0$, we can derive,

(A64) $$\begin{array}{c} \left[-i\frac{\partial }{\partial x^{0}}+eA_{0}+\frac{{\left(\nabla_{i}+ieA_{i}\right)}^{2}}{2m}-\frac{e^{2}D_{ii}^{11}\left(x,x\right)}{2m}\right]{\bar{\varphi }}^{*}\left(x\right)+\frac{1}{2}\frac{\delta \Gamma_{2}}{\delta \bar{\varphi }}=0. \end{array}$$

Using Equations (A63) and (A64), we can derive the conservation law of charge,

(A65) $$\begin{array}{c} \frac{\partial J^{0}}{\partial x^{0}}=\nabla_{i}J_{i}, \end{array}$$

where charge density $J^{0}$ and current $J_{i}$ are given by,

(A66) $$\begin{array}{ccc} J^{0} & = & -e{\bar{\varphi }}^{*}\bar{\varphi }, \end{array}$$

(A67) $$\begin{array}{ccc} J_{i} & = & -\frac{ie}{2m}\left[{\bar{\varphi }}^{*}\left(\nabla_{i}-ieA_{i}\right)\bar{\varphi }-\left(\left(\nabla_{i}+ieA_{i}\right){\bar{\varphi }}^{*}\right)\bar{\varphi }\right]. \end{array}$$

Using the conservation law for coherent fields and Equation (A62), we can derive,

(A68) $$\begin{array}{ccc} \partial^{0}J_{0} & = & -\partial^{i}J_{i}\\ & = & -\partial^{i}\partial^{\nu }F_{\nu i}\\ & = & \partial^{\mu }\partial^{\nu }F_{\nu \mu }-\partial^{i}\partial^{\nu }F_{\nu i}\\ & = & \partial^{0}\partial^{\nu }F_{\nu 0},\;\;\;\to \partial^{\nu }F_{\nu 0}=J^{0}, \end{array}$$

where we have integrated by time $x^{0}$ in the last line, and time-independent constant term interpreted as an initial condition is set to be zero.

We shall rewrite $\bar{\varphi }\left(x\right)=\left|\bar{\varphi }\left(x\right)\right|e^{i\beta (x)}$ and ${\bar{\varphi }}^{*}\left(x\right)=\left|\bar{\varphi }\left(x\right)\right|e^{-i\beta (x)}$ and neglect $\frac{1}{2}\frac{\delta \Gamma_{2}}{\delta {\bar{\varphi }}^{\left(*\right)}}$ terms since they are higher order contributions $O(e^{4}|\bar{\varphi }{|}^{2})$ in the coupling expansion of *e*. We then rewrite the following relations by using imaginary and real part of Equations (A63) and (A64),

(A69) $$\begin{array}{ccc} \frac{\partial }{\partial x^{0}}{\left|\bar{\varphi }\right|}^{2} & = & \frac{e}{m}\nabla_{i}\left[|\bar{\varphi }{|}^{2}\left(A_{i}-\frac{1}{e}\partial_{i}\beta \right)\right], \end{array}$$

(A70) $$\begin{array}{ccc} A_{0}-\frac{1}{e}\partial_{0}\beta & = & -\frac{\nabla_{i}^{2}\left|\bar{\varphi }\right|}{2me|\bar{\varphi }|}-\frac{e}{2m}{\left(A_{i}-\frac{1}{e}\partial_{i}\beta \right)}^{2}, \end{array}$$

where we have neglected $-\frac{e}{2m}D_{ii}^{11}\left(x,x\right)$ term. The Equation (A69) represents conservation law of charge, while Equation (A70) represents constraint of scalar potential $A_{0}$. Furthermore the Equation (A62) is rewritten by,

(A71) $$\begin{array}{ccc} \left[\left(\partial_{0}^{2}-\partial_{k}^{2}\right)\delta_{ij}+\partial_{i}\partial_{j}\right]\left(A_{j}-\frac{1}{e}\partial_{j}\beta \right)-\partial_{i}\partial_{0}\left(A_{0}-\frac{1}{e}\partial_{0}\beta \right)+\frac{e^{2}{\left|\bar{\varphi }\right|}^{2}}{m}\left(A_{i}-\frac{1}{e}\partial_{i}\beta \right) & = & -\frac{1}{2}\frac{\delta \Gamma_{2}}{\delta A^{i}}. \end{array}$$

We find Equations (A69) and (A70) and the left-hand side of Equation (A71) are invariant under gauge transformation $\beta \to \beta +\beta^{\prime }$ and $A_{\mu }\to A_{\mu }+\frac{1}{e}\partial_{\mu }\beta^{\prime }$. Using Equations (A63), (A64), (A69), (A71) and the relation,

(A72) $$\begin{array}{c} \partial_{0}^{2}\left(\partial_{i}\left(A_{i}-\frac{\partial_{i}\beta }{e}\right)\right)=\partial_{0}\left(\partial_{i}^{2}\left(A_{0}-\frac{\partial_{0}\beta }{e}\right)\right)-\frac{e^{2}}{m}\nabla_{i}\left(|\bar{\varphi }{|}^{2}\left(A_{i}-\frac{\partial_{i}\beta }{e}\right)\right), \end{array}$$

which is derived by differentiating Equation (A71) by $x^{i}$, and neglecting quantum fluctuations in the term $\frac{1}{2}\frac{\delta \Gamma_{2}}{\delta A^{i}}$, we can derive the conserved energy *E*,

(A73) $$\begin{array}{ccc} \frac{\partial E}{\partial x^{0}} & = & 0, \end{array}$$

(A74) $$\begin{array}{ccc} E & = & \int d^{3}x[\frac{1}{2}{\left(\partial_{0}\left(A_{i}-\frac{1}{e}\partial_{i}\beta \right)-\partial_{i}\left(A_{0}-\frac{1}{e}\partial_{0}\beta \right)\right)}^{2}\\ & & +\frac{1}{2}\left(\partial_{j}{\left(A_{i}-\frac{1}{e}\partial_{i}\beta \right)}^{2}\right)-\frac{1}{2}{\left(\partial_{j}\left(A_{j}-\frac{1}{e}\partial_{j}\beta \right)\right)}^{2}\\ & & +\frac{1}{2m}{\left(\nabla_{i}\left|\bar{\varphi }\right|\right)}^{2}+\frac{e^{2}}{2m}{\left|\bar{\varphi }\right|}^{2}{\left(A_{i}-\frac{1}{e}\partial_{i}\beta \right)}^{2}]. \end{array}$$

Even in the presence of the terms $-\frac{e}{2m}D_{ii}^{11}\left(x,x\right)$ in Equations (A63) and (A64), and $\frac{1}{2}\frac{\delta \Gamma_{2}}{\delta A^{i}}$ in Equation (A71), we can derive the conserved energy in 2PI formalism by considering time-evolution equations of Green’s functions Δ and $D_{ij}$, namely the Kadanoff–Baym equations, derived by using $\frac{\delta \Gamma_{2\mathrm{PI}}}{\delta \Delta }{|}_{{\bar{a}}_{i}=0}=0$ and $\frac{\delta \Gamma_{2\mathrm{PI}}}{\delta D}{|}_{{\bar{a}}_{i}=0}=0$. The derivation of conserved energy in QED with relativistic charged bosons is in [90]. In a similar way to the case of $\phi^{4}$ model [91], the term $\frac{1}{2}\frac{\delta \Gamma_{2}}{\delta A^{i}}$ can be written by,

(A75) $$\begin{array}{ccc} -\frac{1}{2}\frac{\delta \Gamma_{2}}{\delta A^{i}} & = & \int_{-\infty }^{x^{0}}dy^{0}\Sigma_{\rho }\left(x^{0},y^{0}\right)\left(A_{i}\left(y^{0}\right)-\frac{1}{e}\partial_{i}\beta \left(y^{0}\right)\right)\\ & ≃ & -M^{2}\left(A_{i}\left(x^{0}\right)-\frac{1}{e}\partial_{i}\beta \left(x^{0}\right)\right)-\gamma \partial_{0}\left(A_{i}\left(x^{0}\right)-\frac{1}{e}\partial_{i}\beta \left(x^{0}\right)\right)+O\left(\partial^{2}A\right), \end{array}$$

where $\Sigma_{\rho }$ represents spectral part of self-energy, and $M^{2}$ and γ represent factors dependent on temperature *T* and elementary charge *e*.

We shall consider the case with $A_{3}=0$ and homogeneity of $x^{3}$-direction. Time-evolution Equations (A69)–(A71) with Equation (A75) are rewritten by,

(A76) $$\begin{array}{ccc} \partial_{0}E_{1} & = & \partial_{2}^{2}A_{1}-\partial_{1}\partial_{2}A_{2}-\frac{e|\bar{\varphi }{|}^{2}}{m}A_{1}-M^{2}A_{1}-\gamma \left(E_{1}+\partial_{1}A_{0}\right)+vu_{1}, \end{array}$$

(A77) $$\begin{array}{ccc} \partial_{0}A_{1} & = & E_{1}+\partial_{1}A_{0}, \end{array}$$

(A78) $$\begin{array}{ccc} \partial_{0}E_{2} & = & \partial_{1}^{2}A_{2}-\partial_{1}\partial_{2}A_{1}-\frac{e|\bar{\varphi }{|}^{2}}{m}A_{2}-M^{2}A_{2}-\gamma \left(E_{2}+\partial_{2}A_{0}\right)+vu_{2}, \end{array}$$

(A79) $$\begin{array}{ccc} \partial_{0}A_{2} & = & E_{2}+\partial_{2}A_{0}, \end{array}$$

(A80) $$\begin{array}{ccc} \partial_{0}{\left|\bar{\varphi }\right|}^{2} & = & \frac{e}{m}\left(\partial_{1}\left(|\bar{\varphi }{|}^{2}A_{1}\right)+\partial_{2}\left(|\bar{\varphi }{|}^{2}A_{2}\right)\right), \end{array}$$

(A81) $$\begin{array}{ccc} A_{0} & = & -\frac{\left(\partial_{1}^{2}+\partial_{2}^{2}\right)\left|\bar{\varphi }\right|}{2me|\bar{\varphi }|}+\frac{e(A_{1}^{2}+A_{2}^{2})}{2m}, \end{array}$$

where $E_{1}$ and $E_{2}$ represent electric fields in $x^{1}$ and $x^{2}$ directions, respectively. The *v* represents transmittance between the system and the input layer, and $u_{1}$ and $u_{2}$ represent input functions. Here we omit terms $-\partial_{\mu }\beta /e$ with μ=0,1,2.

Finally we consider the model in a hierarchy involving n=0 layer (input layer) and n=1,2,⋯,N,N+1 layers shown in Figure 3. Time-evolution Equations (A76)–(A81) are extended as,

(A82) $$\begin{array}{ccc} \partial_{0}E_{1}^{\left(n\right)} & = & \partial_{2}^{2}A_{1}^{\left(n\right)}-\partial_{1}\partial_{2}A_{2}^{\left(n\right)}-\frac{e^{2}{\left|{\bar{\varphi }}^{\left(n\right)}\right|}^{2}}{m}A_{1}^{\left(n\right)}-M^{2}A_{1}^{\left(n\right)}-\gamma \left(E_{1}^{\left(n\right)}+\partial_{1}A_{0}^{\left(n\right)}\right)\\ & & +v\left(A_{1}^{\left(n-1\right)}+A_{1}^{\left(n+1\right)}\right), \end{array}$$

(A83) $$\begin{array}{ccc} \partial_{0}A_{1}^{\left(n\right)} & = & E_{1}^{\left(n\right)}+\partial_{1}A_{0}^{\left(n\right)}, \end{array}$$

(A84) $$\begin{array}{ccc} \partial_{0}E_{2}^{\left(n\right)} & = & \partial_{1}^{2}A_{2}^{\left(n\right)}-\partial_{1}\partial_{2}A_{1}^{\left(n\right)}-\frac{e^{2}{\left|{\bar{\varphi }}^{\left(n\right)}\right|}^{2}}{m}A_{2}^{\left(n\right)}-M^{2}A_{2}^{\left(n\right)}-\gamma \left(E_{2}^{\left(n\right)}+\partial_{2}A_{0}^{\left(n\right)}\right)\\ & & +v\left(A_{2}^{\left(n-1\right)}+A_{2}^{\left(n+1\right)}\right), \end{array}$$

(A85) $$\begin{array}{ccc} \partial_{0}A_{2}^{\left(n\right)} & = & E_{2}^{\left(n\right)}+\partial_{2}A_{0}^{\left(n\right)}, \end{array}$$

(A86) $$\begin{array}{ccc} \partial_{0}{\left|{\bar{\varphi }}^{\left(n\right)}\right|}^{2} & = & \frac{e}{m}\left(\partial_{1}\left(|{\bar{\varphi }}^{\left(n\right)}{|}^{2}A_{1}^{\left(n\right)}\right)+\partial_{2}\left(|{\bar{\varphi }}^{\left(n\right)}{|}^{2}A_{2}^{\left(n\right)}\right)\right), \end{array}$$

(A87) $$\begin{array}{ccc} A_{0}^{\left(n\right)} & = & -\frac{\left(\partial_{1}^{2}+\partial_{2}^{2}\right)\left|{\bar{\varphi }}^{\left(n\right)}\right|}{2me|{\bar{\varphi }}^{\left(n\right)}|}+\frac{e\left({\left(A_{1}^{\left(n\right)}\right)}^{2}+{\left(A_{2}^{\left(n\right)}\right)}^{2}\right)}{2m}, \end{array}$$

with n=1,2,⋯,N.

We adopt morphological computation using input–output equations [62] in developing the control theory in holographic brain theory. We shall set target functions $u_{1}^{\left(N\right)}=A_{1,\mathrm{target}}$ and $u_{2}^{\left(N\right)}=A_{2,\mathrm{target}}$ with $u_{1}^{\left(N+1\right)}=u_{2}^{\left(N+1\right)}=0$. The input functions $u_{1}^{\left(0\right)}$ and $u_{2}^{\left(0\right)}$ are calculated by,

(A88) $$\begin{array}{ccc} u_{1}^{\left(N-J\right)} & = & \frac{-\partial_{1}^{2}u_{1}^{\left(N-J+1\right)}+\partial_{1}\partial_{2}u_{2}^{\left(N-J+1\right)}+M^{2}u_{1}^{\left(N-J+1\right)}}{v}-u_{1}^{\left(N-J+2\right)}, \end{array}$$

(A89) $$\begin{array}{ccc} u_{2}^{\left(N-J\right)} & = & \frac{-\partial_{2}^{2}u_{2}^{\left(N-J+1\right)}+\partial_{1}\partial_{2}u_{1}^{\left(N-J+1\right)}+M^{2}u_{2}^{\left(N-J+1\right)}}{v}-u_{2}^{\left(N-J+2\right)}, \end{array}$$

with J=1,2,⋯N. The $u_{1}^{\left(N-1\right)}$ in Equation (A88) is derived by substituting $A_{1}^{\left(N\right)}$ by $u_{1}^{\left(N\right)}$, $A_{1}^{\left(N+1\right)}$ by $u_{1}^{\left(N+1\right)}$, and $A_{1}^{\left(N-1\right)}$ by $u_{1}^{\left(N-1\right)}$ in Equation (A82) and neglecting terms with time-derivatives and the term $\frac{e^{2}{\left|{\bar{\varphi }}^{\left(N\right)}\right|}^{2}}{m}u_{1}^{\left(N\right)}$. The $u_{1}^{\left(N-2\right)}$ is derived by substituting $A_{1}^{\left(N-1\right)}$ by $u_{1}^{\left(N-1\right)}$, $A_{1}^{\left(N\right)}$ by $u_{1}^{\left(N\right)}$, and $A_{1}^{\left(N-2\right)}$ by $u_{1}^{\left(N-2\right)}$ in Equation (A82) and neglecting terms with time-derivatives and the term $\frac{e^{2}{\left|{\bar{\varphi }}^{\left(N-1\right)}\right|}^{2}}{m}u_{1}^{\left(N-1\right)}$. Repeat until $u_{1}^{\left(0\right)}$ is derived. Similarly, we derive $u_{2}^{\left(0\right)}$ by Equation (A89). Finally, we set

(A90) $$\begin{array}{ccc} A_{1}^{\left(0\right)} & = & u_{1}^{\left(0\right)}\exp\left(-\gamma_{2}x^{0}\right), \end{array}$$

(A91) $$\begin{array}{ccc} A_{2}^{\left(0\right)} & = & u_{2}^{\left(0\right)}\exp\left(-\gamma_{2}x^{0}\right), \end{array}$$

with damping factor $\gamma_{2}$ for input functions $A_{1}^{\left(0\right)}$ and $A_{2}^{\left(0\right)}$.
